# Emotional intelligence as a crucial component to medical education

**DOI:** 10.5116/ijme.5654.3044

**Published:** 2015-12-06

**Authors:** Debbi R. Johnson

**Affiliations:** 1 St. George’s University, True Blue Campus, St. George’s, Grenada, West Indies

**Keywords:** Emotional intelligence, medical education, professionalism, graduate education

## Abstract

**Objectives:**

The primary focus of this review was to discover what is already known about Emotional Intelligence (EI) and the role it plays within social relationships, as well as its importance in the fields of health care and health care education. This article analyzes the importance of EI in the field of health care and recommends various ways that this important skill can be built into medical programs.

**Methods:**

Information was gathered using various database searches including EBSCOHOST, Academic Search Premier and ERIC. The search was conducted in English language journals from the last ten years. Descriptors include: Emotional Intelligence, medical students and communication skills, graduate medical education, Emotional Intelligence and graduate medical education, Emotional Intelligence training programs, program evaluation and development.

**Results:**

Results of the study show a direct correlation between medical education and emotional intelligence competencies, which makes the field of medical education an ideal one in which to integrate further EI training.

**Conclusions:**

The definition of EI as an ability-based skill allows for training in specific competencies that can be directly applied to a specialized field. When EI is conceptualized as an ability that can be taught, learned, and changed, it may be used to address the specific aspects of the clinician–patient relationship that are not working well. For this reason, teaching EI should be a priority in the field of medical education in order to better facilitate this relationship in the future.

## Introduction

The goals and objectives of education, and therefore, educators, have evolved in recent years. Higher education traditionally has focused on a subject-oriented perspective; however, the knowledge base is rapidly changing, so in addition to mastering content, the learner must master the ability to continue to learn as a self-directed and lifelong learner. These constant advancements certainly have affected the field of medical education, where teachers have the responsibility of helping students to approach material from a more consumer-oriented perspective, giving them the skills to become lifelong learners and mentors. Consequently, every effort should be made by educators to "move the learners gradually but firmly in the direction of autonomy and self-directedness".^1^

In a study of medical students in both academic and nonacademic difficulty at George Washington University, Hendren (1988) identified four major groups of student issues that affected attrition: (a) intrapersonal issues, (b) interpersonal issues, (c) academic problems, and (d) a combination of extreme anxiety and limited academic ability.^2^ Hendren's study was important as it identified reasons other than simply academic ability as key factors in student success.

Students with intrapersonal problems can be defined as those who struggle due to personal internal conflicts or anxiety.^2^ Results of a study conducted at the University of Alberta showed a higher level of stress and depression among students in the health sciences than among other graduate students.^3^ Reasons for this additional stress included greater competition among students in these fields and these programs' responsibility for graduating knowledgeable and skilled professionals who will be performing in often-stressful conditions. It must be noted, however, that although the curriculum is geared toward fostering the greatest amount of learning possible, some aspects of training may negatively affect the student's health.^4^ Whereas a certain degree of anxiety is useful for performance, to an excess it can become debilitating and lead to many other problems.^2^ The competitive nature of the health sciences, combined with certain academic weaknesses, serves to create poorly adaptive perfectionism in students within these programs that leads to unrealistic and excessive concerns about performance.^3^

The second area identified by Hendren (1988) was interpersonal interactions among students or between students and their teachers. Students with interpersonal issues are identified as those who have difficulties interacting with professors, colleagues, clinicians, and ultimately patients. These students tend to suffer both academically and nonacademically over the course of their medical education in all subjects, although they tend to have greater difficulties during their clinical years.^2^ Although they may be capable of coping with the traditional approach to medical education, which focuses on individual rather than group learning, they are unable to succeed with the new approach requiring interactive learning and participation.^5^ It is the purpose of this paper to conduct a review of the literature regarding the teaching of these interpersonal skills through Emotional Intelligence training and the importance that it serves in Medical Education.

## Methods

Information was gathered using various database searches including EBSCOHOST, Academic Search Premier and ERIC. The search was conducted in English language journals from the last ten years. Descriptors included: Emotional Intelligence, medical students and communication skills, graduate medical education, Emotional Intelligence and graduate medical education, Emotional Intelligence training programs, program evaluation and development. The primary focus of the literature review was to discover what is already known about EI and the role it plays in the field of health care and health care education. Subsequently, a determination was made regarding which skills, procedures and tasks are required to build these competencies into a program focused specifically on health care education, and recommendations were made as to its implementation.

## Results

The competency of communication in particular is an important component of the expectations for learning and assessment that the Accreditation Council for Graduate Medical Education.^6^ provided and also falls under the umbrella of Emotional Intelligence (EI). Grewal and Davidson (2008) proposed that the scientific model of EI serves to better help students understand the Accreditation Council for Graduate Medical Education competency of professionalism, which involves strong interpersonal and communication skills. This EI theory can help better understand the complexities of interpersonal competencies as well as how to better integrate these skills into graduate medical training.

Intelligence is a concept that can be defined in various ways, such as dependent on memories or processes, verbal intelligence, or spatial intelligence. The traditional classification of intelligences divides them into (a) verbal and propositional and (b) perceptual and organizational areas, although for decades investigators have been searching for the hard-to-define third intelligence that adequately accounts for individual differences in intelligence.^7^ Early definitions of social intelligence alluded to these differences in a person's ability to successfully manage human relationships, but it was not until the early 1980s that the idea of multiple intelligences began to surface.^8^ During this same time, research on emotions was growing, most notably investigations into Darwin's ideas regarding the universality of expression of emotion.^9^ As emotions convey such valuable information regarding individuals and their relationships with others, the effective understanding and managing of emotions is now considered a type of intelligence. Mayer and Salovey (1993) highlighted the difference between personality traits and EI, in that the former may rely on various social skills or preferences, "whereas truly knowing what a person feels is a mental ability".^10^ The theory of EI is based on several key ideas. From the field of intelligence it takes the idea that intelligence must include the ability to think abstractly. From research on emotions it takes that emotions are signals that give meaning regarding relationships. Another key idea is that many emotions can be considered universal in that they are recognized through basic emotional expressions across various cultures.

The term EI best describes a great variety of non-cognitive qualifications and competencies that help individuals cope with environmental demands and stressors. Brannick, Wahi, Arce, Johnson, Nazian and Goldin (2009) defined EI as "the ability to perceive emotions, to access and generate emotions so as to assist thought, to understand emotions and emotional knowledge, and to reflectively regulate emotions so as to promote emotional and intellectual growth".^11^ The concept of EI has received a significant amount of media attention, particularly with the publication of Goleman's (1995) book, Emotional Intelligence: Why it Can Matter More Than IQ for Character, Health and Lifelong Achievement.^12^ This work received a great deal of exposure in the press, including a cover on Time magazine, which launched the concept of EI into popular culture.^7^ Goleman's work, however, was based on Salovey and Mayer's (1990) four-branch model of EI, which was proposed as a way to better explain the psychological differences in individuals' abilities related to emotion. Salovey and Mayer contended that EI is a set of abilities that allows individuals to engage in complex processing regarding their own and others' emotions, as well as to use that information to guide thoughts and behavior. To this end, individuals with high EI are able to use, understand, and manage emotions effectively, which works to their own and others' benefit.^13^The first branch of EI, perceiving emotions, is the fundamental skill of EI.^14^^,^^15^ This branch represents the ability to perceive and identify one's own emotions as well as those of others, and to acknowledge emotions brought about from objects, art, stories, music, and various other stimuli. The second branch of EI, using emotions, involves the ability to generate and use emotions best suited to particular activities. According to Salovey and Grewal (2005), an individual skilled in this area is able to make the most of changing emotions in order to best accomplish tasks.15 The third branch, understanding emotions, includes the ability to understand and verbalize in a somewhat sophisticated manner both the existence of emotions and the complex ways they work together. Additionally, this branch involves the ability to understand the natural development of emotions over time. The fourth branch of EI is managing emotions, the ability to regulate and manage emotions in ourselves and others.^15^ An individual skilled in this area can manage his or her own emotions in explicit ways to meet specific goals. Out of these four branches, emotion regulation is the most complex yet most important for appropriate social interaction because it directly impacts the way an individual will express themselves and behavior.^16^

## Discussion

### Emotional intelligence and medical education

Although researchers who initially claimed that EI can be more important in determining success than IQ have since minimized their own claims.^7^ there is no question that it plays a significant role. Numerous authors have asserted that EI contributes to an individual's ability to adapt socially, work more effectively in teams, perform better, and cope more effectively with stress and other forms of environmental pressure.^14^^,^^17^^-^^19^ Furthermore, college students with high scores on the managing-emotions subscale of the MSCEIT described having less conflict in their relationships with both colleagues and superiors.^16^

 In the past, traditional medicine has encouraged health care providers to preserve an emotional distance from their patients in order to maintain a certain degree of objectivity.^20^ In recent years, however, there has been a significant move toward breaking down barriers of communication between patients and health care practitioners, in favor of a more empathic approach. As the relationship between patients and health care providers becomes more of a partnership, fostering good communication skills in order to improve patient satisfaction and build mutual understanding becomes a major focus in the field of medicine.

When considering the quality of the health care practitioner - patient relationship, EI is a fundamental component of effective practice and is generating increased interest in the field of health care.^11^ From hospital administrators to physicians and nurses, collaboration is needed not only to improve cost effectiveness of practice but also to ensure patient compliance and satisfaction. Patient autonomy, allowing medical consultations to be patient led, is another integral part of positive interactions between health care practitioners and patients. In order for this dynamic to occur, the health care provider must be competent in EI and able to recognize shifts in a patient's moods and demeanor. The patient-centered approach is a holistic one that acknowledges a patient's needs for complete information and active participation in diagnosis, treatment, and long-term adherence. Given that the majority of criticisms about health care practitioners relate to poor communication skills, proficiency in the area of EI undoubtedly could improve patient satisfaction and concordance.^25^ Capable assessment of a patient's emotions would have an immediate effect on the accuracy of history taking and diagnosis. Furthermore, if a clinician understands the patient's background and emotional reactions, the medical advice and treatment given can be tailored to match the individual's expectations.

### Teaching emotional intelligence

When considering whether or not EI can be taught, several issues must be taken into account. First, it is necessary to establish a likely rationale for the process required to learn EI. Second, the effectiveness of evidence regarding attempts to teach EI must be considered. Third, given that educational environments are complex and dynamic, the various factors related to successful implementation of EI programs must be explored.^22^

With regards to the process of emotional learning, it is necessary only to consider neuro-anatomical function. According to Humphrey et al. (2007), EI represents "the ability of the higher brain centers to monitor and direct more primitive emotional signals from phylogenetically older brain structures, such as the amygdala, in such a way that they are used constructively by the individual, rather than destructively".^22^ The amygdala is the more primitive of structures that controls more basic and self-centered impulses and is subject to control from higher cortical structures such as the frontal lobes. Individuals exhibiting higher levels of EI likely are able to identify emotional states in themselves and others using these higher cortical structures. These individuals then can take this information and use it in ways that best suit the environment they are in, controlling it according to each respective situation. Therefore, education of EI provides the higher brain centers with new ways to understand and respond to the environment. In a sense, it teaches the individual to move from behaviors seeking self-gratification to ones where gratification is received by understanding emotional needs in the self and others.

Given the sensitive nature of emotions, EI must be taught in an environment where students feel supported in order for it to be successful and sustainable.^20^ Furthermore, the program must be appropriately targeted for the right sociocultural context as well as the specific school culture. The programs must endeavor to promote an appreciation for diversity and must be "sensitive, relevant, and responsive with regard to the ethnic, gender and socioeconomic composition of the students".^21^ Along with the creation of an appropriate context for the teaching of EI, it is important that the material be age appropriate and integrated into the school's educational curriculum in order to increase buy-in. This is particularly necessary when dealing with students at the graduate level and specifically in the rigorous field of health care.

## Conclusions

When working with graduate students to address EI, learning outcomes must be precise when including them in a program.^20^ One major reason is that many individuals in health care fields feel that they entered into the field out of a sense of compassion for the suffering of others. They also have the misconception that this feeling of compassion is all that is required to connect with their patients and that it will be a constant throughout their careers. Consequently, they may not see the value of EI training, and programs directed toward addressing these competencies must be well structured, with explicit objectives and clear purpose.^20^^,^^21^

The implementation of EI-development programs in schools does require acceptance of few basic assumptions. First, schools will be supporting the development throughout the academic year, not simply for short periods. Second, continuous development of EI skills is assumed to help students cope with the pressures of their respective environments. Finally, EI must be addressed collaboratively by the students, faculty and administration in any given program, and everyone must be on board.^21^^,^^22^ Zeidner et al. (2002) identified seven characteristics that EI-development programs must contain in order to be successful: (a) a working definition of EI, as different interpretations would lead to different types of interventions; (b) clear objectives and outcome expectations; (c) clear identification of the educational context in which the program will take place; (d) full integration of the EI program into the curriculum, (e) work with EI in context that directly applies to the field; (f) development of staff involved in teaching; and (g) appropriate psychometrically sound evaluation of the EI program being implemented.^21^

The definition of EI as an ability-based skill allows for training in specific competencies that can be directly applied to a specialized field.^14^^, ^^21^ When EI is conceptualized as an ability that can be taught, learned, and changed, it may be used to address the specific aspects of the clinician - patient relationship that are not working well.^23^ Therefore, in addressing the first necessary characteristic for successful interventions, defining EI using the Mayer and Salovey (1997) model is most useful for specialized purposes.^25^

To identify program goals for the target population, special consideration must be given to addressing EI competencies within the conceptual framework of each specific program. All materials and components must be carefully chosen to meet the needs of the program they are being created for. This careful selection is particularly important in graduate programs, where students have a challenging curriculum and are less likely to buy in to extra material that they consider irrelevant. In addition to meticulous selection of materials, careful consideration must be given to the sociocultural context in which the training is to take place, in order for it to be most appropriate. According to Zeidner, Roberts and Matthews (2002), the relevant population characteristics that need to be identified from the beginning include student age, school environment and culture, teacher and administrative staff characteristics, and characteristics of the broader community.^21^ Programs should provide developmentally appropriate training that progresses as the students continue through the coursework.

Ideally, EI skills would not be developed in workshops or seminars that are added on to an already-full curriculum. Students must see EI as an integral part of their existing program and not as something to be developed in a separate environment. Furthermore, in order to produce meaningful results, students must be given opportunities to practice these skills in contexts similar to the ones they will be facing outside the classroom. EI training should be interdisciplinary and holistic, an opportunity for education and leadership development through practical, applied preparation.^21^^,^^23^

EI training is not typically part of the standard curriculum for educators, and thus many teachers feel uncomfortable incorporating it into their classes. Therefore, programs that have EI training incorporated into the curriculum also must have means to provide staff with adequate knowledge and skills before, during, and after program implementation. For this acquisition of EI knowledge to occur, professional development programs for faculty and staff must be running parallel to EI skills training in order to provide necessary support. Finally, EI programs must have clearly established ways to implement, monitor, and evaluate the training. If the quality of delivery and implementation is not evaluated, it is difficult to accurately assess and replicate program outcomes.^21^

Undoubtedly, there are guidelines for the implementation of EI interventions, although they are more generic in nature, rather than targeted to a specific population. Although little information exists about how EI skills should be most effectively taught, the concept of EI has proven to be the impetus needed for educators to begin to consider how it affects performance and success. The school setting is the perfect context for learning emotional competencies in a safe environment and developing skills in areas related to their respective fields.^22^ As EI competencies are particularly important in the field of health care, it is exceptionally important for graduate programs in the field to encourage students to increase their skills in these areas.^11^

Austin, Evans, Magnus and O' Hanlon (2007) showed that cognitive intelligence is neither the only factor in the making of a successful health worker nor the only predictor of leadership success.^25^ Given significant importance placed on the quality of medical care, EI of health care practitioners has been the subject of increasing interest.^11^ Clearly, all of these qualities are crucial to successful clinicians. Characteristics of EI are similar to personality traits but can be altered and improved through effort on the part of the individual. Therefore, the skills associated with EI, such as perceiving, understanding, using, and managing emotions, can be improved through training that specifically addresses these skills.

### Conflict of Interest

The authors declare that they have no conflict of interest.
